# Supplementary material for: Single component CRISPR-mediated base-editors for Agrobacterium and their use to develop an improved suite of strains
Source: Biodes Res. 2025 Feb 27;7(1):100001. doi: 10.1016/j.bidere.2025.100001 (PMC12709902; doi:10.1016/j.bidere.2025.100001)
Supplement: Multimedia component 2 [file mmc2.docx]

**Supplementary tables**

Supplementary Table 1 List of primers used in this study.

| Sequence ID | Oligos (5' → 3') | Description |
| --- | --- | --- |
| dCas9 F | TCCAAGCTCAAGCTAAGCTTCGAGCATTTTCGTTCTGAGCC | Gibson assembly forward primer for amplification of Target-AID editor cassette from pEN-L4-PvirB-dCas9-CDA-UL-T3T-R1 |
| dCas9 R | TAGCTGTCAATGCAAAAAACCCCTCAGACTC | Gibson assembly reverse primer for amplification of Target-AID editor cassette from pEN-L4-PvirB-dCas9-CDA-UL-T3T-R1 |
| sfGFP F | GTTTTTTGCATTGACAGCTAGCTCAGTCCT | Gibson assembly forward primer for amplification of sGFP-gRNA scaffold cassette from pEN-L1-PJ23119-Bsa1-PglpT-sfGFP-TrrfB-Bsa1-Scaf-L2 |
| sfGFP R | TGTGCACCTGAAAAAAAGCACCGACTCGGTG | Gibson assembly reverse primer for amplification of sGFP-gRNA scaffold cassette from pEN-L1-PJ23119-Bsa1-PglpT-sfGFP-TrrfB-Bsa1-Scaf-L2 |
| sacB F | TGCTTTTTTTCAGGTGCACATATACCTGCC | Gibson assembly forward primer for amplification of *sacB* cassette from pEN-R2-SacB-L3 |
| sacB R | CCGACGTCGCATGCCTGCAGACACTCGCATACGCGATTCT | Gibson assembly reverse primer for amplification of *sacB* cassette from pEN-R2-SacB-L3 |
| K599_ThyA_gtype F | CGCCTTGATGCTTGAATCG | Forward genotyping primer for *thyA* knockout in *A. rhizogenes* strain K599 |
| K599_ThyA_gtype R | TCATCCATGAATTGCTGTGG | Reverse genotyping primer for *thyA* knockout in *A. rhizogenes* strain K599 |
| K599_RecA_gtype_F | CACCGGTCTTGGAAACGC | Forward genotyping primer for *recA* knockout in *A. rhizogenes* strain K599 |
| K599_RecA_gtype_R | GAAATTTACGGCCCGGAAAG | Reverse genotyping primer for *recA* knockout in *A. rhizogenes* strain K599 |
| EHA105_thyA_gtype_F | CTCCAGCGTAAAGTCCTCG | Forward genotyping primer for *thyA* in the C58 background |
| EHA105_thyA_gtype_R | TGGTTCCTAAACGGCGATAC | Reverse genotyping primer for *thyA* in the C58 background |
| EHA105_recA_gtype_F | GCCATACATGATGTCGAATTCAAC | Forward genotyping primer for *recA* in the C58 background |
| EHA105_recA_gtype_R | GCGCTGCAGACGATCG | Reverse genotyping primer for *recA* in the C58 background |
| Ach5_thyA_gtype_R | GGAGTTCATGGATGATCGAG | Forward genotyping primer for *thyA* in the Ach5 background |
| Ach5_thyA_gtype_F | GAGAAGAGGTCTCGTACATC | Reverse genotyping primer for *thyA* in the Ach5 background |
| Ach5_recA_gtype_F | TACATGATGTCGAATTCCACC | Forward genotyping primer for *recA* in the Ach5 background |
| Ach5_recA_gtype_R | CTTCCGCGATCGTCTGCA | Reverse genotyping primer for *recA* in the Ach5 background |
| EHA105_thyA_guide | TCCTAGGTATAATGCTAGCTTCCAGTTCTATGTGTCCGAGTTTTAGAGCTAGAAATAG | Gibson assembly oligo for sgRNA targeting *thyA* Q151* in the C58 background |
| EHA105_recA_guide | TCCTAGGTATAATGCTAGCTCAGGCCCGTCTGATGAGCCGTTTTAGAGCTAGAAATAG | Gibson assembly oligo for sgRNA targeting *recA* Q178* in the C58 background |
| Ach5_recA_guide | TCCTAGGTATAATGCTAGCTCAGGCACGTCTGATGAGCCGTTTTAGAGCTAGAAATAG | Gibson assembly oligo for sgRNA targeting *recA* Q178* in the Ach5 and chry5 backgrounds |
| k599thyA2 | TCCTAGGTATAATGCTAGCTTCCAGTTCTATGTTTCCGAGTTTTAGAGCTAGAAATAG | Gibson assembly oligo for sgRNA targeting *thyA* Q151* in the K599 background |
| Ach5thyA2 | TCCTAGGTATAATGCTAGCTTCCAGTTCTATGTTTCGGAGTTTTAGAGCTAGAAATAG | Gibson assembly oligo for sgRNA targeting *thyA* Q151* in the Ach5 and chry5 backgrounds |
| chry5_long_RF_R | GTATATGTGCACCTGGATGTGCGCTCCGAAAAAGTGCC | Reverse Gibson assembly primer for cloning the flank 3' to the right T-DNA in pTiChry5 |
| chry5_long_LF_F | ATCTGTTGTTTAAGCTCCCCATCTTAAAACGAGAAAAATGC | Forward Gibson assembly primer for cloning the flank 5' to the left T-DNA in pTiChry5 |
| chry5_D_LF_R | AGGAACTTCGGAGCCTGGCGATTGCCAGTTTATTTGTC | Reverse Gibson assembly primer for cloning the flank 5' to the left T-DNA in pTiChry5 |
| chry5_F_RF_F | ATAGGAACTTCTCAGATGGGAAGTTGGAATGTCCG | Forward Gibson assembly primer for cloning the flank 3' to the right T-DNA in pTiChry5 |
| chry5_3167F | GAACATCGGCTGGAATTCGC | Forward genotyping primer for the T-DNA region on pTiChry5 |
| chry5_6290R | GTTTCATCAGCCGCGGTTAC | Reverse genotyping primer for the T-DNA region on pTiChry5 |
| C5_sf_F | GAATTCGCGGCCGCTTCTAGAGGTGTAGGCTGGAGCTGCTTCG | Forward Gibson assembly primer for amplifying the FRT flanked aada1/sfGFP reporter for assembly with the pTiChry5 disarming flanks |
| C5_sf_R | GACATTCCAACTTCCCATCCCAGTTCCTATTCCGAAGTTC | Reverse Gibson assembly primer for amplifying the FRT flanked aada1/sfGFP reporter for assembly with the pTiChry5 disarming flanks |
| TJBE_HDB_F | GCCTTTCGTTTTATCTGTTGTTTACGAGCATTTTCGTTCTGAGCC | Forward Gibson assembly primer for moving the editor machinery into VPHDB-based chromoprotein-marked backbones |
| TJBE_HDB_R | ATGCGAGTGTGGCACGAACCCAGTGGACATAAGCC | Reverse Gibson assembly primer for moving the editor machinery into VPHDB-based chromoprotein-marked backbones |
| LBA-thyA14F | TCGATCTTCTCCGGCATGTG | Alternate forward genotyping primer for *thyA* in the Ach5 background, compatible with Chry5 |
| LBA-thyA779R | GCCTTGATGGTTGAATCCGC | Alternate reverse genotyping primer for *thyA* in the Ach5 background, compatible with Chry5 |
| LBA-recA_46F | AAAAGCAAGGCACTGGAAGC | Alternate forward genotyping primer for *recA* in the Ach5 background, compatible with Chry5 |
| LBA-recA_1035R | GAACCGATCGGCGATCAGAC | Alternate reverse genotyping primer for *recA* in the Ach5 background, compatible with Chry5 |
| K599_recAg2 | TCCTAGGTATAATGCTAGCAACCAGATCCGCATGAAGATGTTTTAGAGCTAGAAATAG | Gibson assembly oligo for sgRNA targeting *recA* Q205* in the K599 background |

Supplementary Table 2 List of assemblies used in this study.

| Shorthand | Verbose | Description |
| --- | --- | --- |
| pVP073 | pVPHDB-Se | eforRed marked spectinomycin-resistant plasmid backbone with the *sacB* counterselection marker |
| pVP074 | pVPHDB-Ke | eforRed marked kanamycin-resistant plasmid backbone with the *sacB* counterselection marker |
| pVP075 | pVPHDB-KAe | AeBlue marked kanamycin-resistant plasmid backbone with the *sacB* counterselection marker |
|  | pGGPK-AG2 | Kanamycin derivative of pGGP-AG. Pennetti et al., 2024 |
|  | pGGP-AG | Decaestecker et al., 2019 |
| pVP141 | pSpCas9d-CDA1-sfGFP-SacB-GGPK | Single component base-editor vector, kanamycin resistance, no chromoprotein marker, T-DNA borders intact |
| pVP143 | pSpCas9d-CDA1-sfGFP-SacB-KAe | Single component base-editor vector, kanamycin resistance, AeBlue chromoprotein marker, T-DNA borders deleted |
| pVP144 | pSpCas9d-CDA1-sfGFP-SacB-Se | Single component base-editor vector, spectinomycin resistance, eforRed chromoprotein marker, T-DNA borders deleted |
| RskNo4 | pGmUbi3::RUBY::AtHspT-StUbI-3P::spec:StUbi-3T | Dicot RUBY reporter expression plasmid with spectinomycin plant selectable marker produced through GreenGate cloning |
| pVP097 | p143_K599_thyAg2 | pVP143-derived editor plasmid targeting *thyA* Q151* in K599 backgrounds |
| pVP098 | p143-LBA-thyA2 | pVP143-derived editor plasmid targeting *thyA* Q151* in Ach5 and chry5 backgrounds |
| pVP099 | p143-EHA-thyA | pVP143-derived editor plasmid targeting *thyA* Q151* in C58 backgrounds |
| pVP124 | p143-EHA-RecA | pVP143-derived editor plasmid targeting *recA* Q178* in C58 backgrounds |
| pVP125 | p143-K599-RecA | pVP143-derived editor plasmid targeting *recA* Q205* in K599 backgrounds |
| pVP149 | p144-ach5-thyag2 | pVP144-derived editor plasmid targeting *thyA* Q151* in Ach5 and chry5 backgrounds |
| pVP159 | p143-K599-recA2 | pVP143-derived editor plasmid targeting *recA* Q205* in K599 backgrounds |
| pVP210 | p144-ach5-recA | pVP144-derived editor plasmid targeting *recA* Q178* in ach5 and chry5 backgrounds |
| pVP237 | p144-k599thyAg2 | pVP144-derived editor plasmid targeting *thyA* Q151* in K599 backgrounds |
| pVP238 | p144-EHAthyA | pVP144-derived editor plasmid targeting *thyA* Q151* in C58 backgrounds |
